# Prevalence of pectinate ligament dysplasia and associations with age, sex and intraocular pressure in the Basset hound, Flatcoated retriever and Dandie Dinmont terrier

**DOI:** 10.1186/s40575-016-0033-1

**Published:** 2016-03-12

**Authors:** James A. C. Oliver, Abel Ekiri, Cathryn S. Mellersh

**Affiliations:** Centre of Preventive Medicine, Animal Health Trust, Lanwades Park, Kentford, Newmarket, Suffolk, CB8 7UU UK

**Keywords:** Goniodysgenesis, Pectinate ligament dysplasia, Primary closed angle glaucoma

## Abstract

**Background:**

The aims of this study were to: determine the prevalence of pectinate ligament dysplasia (PLD) in populations of Basset hounds (BH), Flatcoated retrievers (FCR) and Dandie Dinmont terriers (DDT) resident in the UK; investigate possible associations between the degree of PLD and age, sex and intraocular pressure (IOP) and; investigate possible associations between IOP and age and sex. Gonioscopy was performed in both eyes of 198 BH, 170 FCR and 95 DDT and the percentage of iridocorneal angle affected by PLD was estimated and classified as unaffected (0 %), mildly affected (<20 %), moderately affected (20–90 %) or severely affected (>90 %). Rebound tonometry was performed bilaterally in the majority of enrolled dogs.

**Results:**

Seventy-six of 198 (38.4 %) BH, 36/170 (21.2 %) FCR and 21/95 (22.1 %) DDT were moderately or severely affected by PLD. The prevalence of PLD was significantly higher in BH than both FCR and DDT. In all breeds there was a significant positive correlation between PLD and age. In the BH only there was a significant association between PLD and sex. In the DDT only there was a weak negative correlation between PLD and IOP and a moderately strong negative correlation between IOP and age.

**Conclusions:**

PLD is prevalent and significantly associated with age in all three breeds we investigated. The linear relationship between PLD and age can be explained by the progression of PLD over time which would contribute to the high prevalence of PLD despite widespread screening.

## Background

The canine ‘glaucomas’ constitute a diverse group of diseases which share the risk factor of elevated intraocular pressure (IOP) and the consequence of progressive degeneration of retinal ganglion cells and the optic nerve. Glaucoma is termed *primary* when it is not a result of identifiable antecedent intraocular disease and can be further classified on the basis of the appearance of the iridocorneal angle (ICA) as seen on gonioscopy. In primary open angle glaucoma (POAG), the ICA appears normal before the onset of disease. However, in primary closed angle glaucoma (PCAG), an abnormality in the structure of the ICA, termed pectinate ligament dysplasia (PLD), is usually apparent before glaucoma develops [1]. PLD is reported be inherited and significantly associated with PCAG in several breeds including the Basset hound, Flatcoated retriever, Dandie Dinmont terrier, English springer spaniel, Welsh springer spaniel and Samoyed [1–7].

In man, females are affected by PCAG more frequently than males [8–10]. The association between sex and canine PCAG is less clear although female American Cocker spaniels, Cocker spaniels, Basset hounds, Welsh springer spaniels and Samoyeds have been reported to be affected more frequently with PCAG than males [2, 11]. A sex difference has also been described for PLD in the American Cocker spaniel but not in the English springer spaniel, Flatcoated retriever or Samoyed [3–5, 12]. Age-related narrowing of the canine ICA has already been documented in more than one breed and recently progression of PLD over time in a single breed over time been has been reported [5, 13, 14]. In a study of 96 Flatcoated Retrievers, 39 (40.6 %) demonstrated progression of PLD with 12/96 dogs (12.5 %) demonstrating progression to severe PLD and being considered at risk of glaucoma development [13]. Furthermore, a recent study reported a positive correlation between age and PLD severity which provides cross-sectional evidence of PLD progression over time [15]. Intraocular pressure (IOP) does not appear to be influenced by sex or degree of goniodysgenesis/PLD in any of the canine breeds investigated thus far [3, 5, 15].

The aims of this study were to estimate the prevalence of PLD in Basset hounds (BH), Flatcoated retrievers (FCR) and Dandie Dinmont terriers (DDT) resident in the UK and to investigate possible associations between PLD and sex and age, and between IOP and sex and age in these three breeds. We hypothesized that there would be no associations between any of these variables.

## Results

### Basset hound (BH)

Of the 198 BH examined, 72 (36.4 %) were male and 126 (63.6 %) were female*.* The median age of BH was 43.07 months (minimum = 3.97 months, maximum = 152.87 months). One hundred forty five of 198 (73.2 %) of BH were affected by PLD (ordinal grades 1–3); 69 (34.8 %) mildly affected (grade 1), 72 (36.4 %) moderately affected (grade 2), and 4 (2 %) severely affected (grade 3) (Table 1). A significant positive correlation was observed between PLD and age (rho = 0.26, *P* < 0.01). No correlation was observed between PLD and IOP (rho = 0.14, *P* = 0.06). The proportion of female dogs affected by PLD (grades 1–3) was higher compared to male dogs (*P* = 0.004). The normality test revealed that the variables IOP (*P* < 0.001) and age (*P* < 0.001) were not normally distributed and as such, the relationship between IOP and age was examined using Spearman’s correlation coefficient. No correlation was observed between IOP and age (rho = 0.043, *P* = 0.55). Average IOP was not significantly different between male and female dogs (*P* = 0.53).

**Table 1:** Frequency of PLD/percentage of ICA circumference affected in both eyes of BH, FCR and DDT dogs

PLD grade^a^	BH^b^			FCR^b^			DDT^b^		
	N	%	95 % CI	N	%	95 % CI	N	%	95 % CI
0	53	26.8	20.6, 32.9	64	37.6	30.4, 44.9	43	45.3	35.3, 55.3
1	69	34.8	28.2, 41.5	70	41.2	33.8, 48.6	31	32.6	23.2, 42.1
2	72	36.4	29.7, 43.1	36	21.2	15.0, 27.3	20	21.1	12.9, 29.3
3	4	2	0.1, 3.9	0	0		1	1.1	0.0, 3.1
Total	198			170			95		

^a^ Ordinal grade for degree of PLD estimated by gonioscopy as percentage of total ICA circumference affected

^b^ BH, FCR and DDT dogs: The mean percentage of ICA affected by PLD for the left and right eye were averaged and then assigned an ordinal score (0, 1, 2, 3)

### Flatcoated retriever (FCR)

Of the 170 FCR examined, 67 (39 %) were male and 103 (61 %) were female. The median age of FCR dogs examined was 48.25 months (minimum = 7.5 months, maximum = 147.6 months). One hundred and six of 170 (62.4 %) FCR were affected by PLD (ordinal grades 1–3); 70 (41.2 %) being mildly affected (grade 1), 36 (21.2 %) moderately affected (grade 2) and 0 severely affected (grade 3) (Table 1). A significant positive correlation was observed between PLD and age (rho = 0.34, *P* < 0.01). No correlation was observed between PLD and IOP (rho = −0.02, *P* = 0.85). The proportion of dogs affected by PLD (grades 1–3) was not significantly different between male and female dogs (*P* = 0.34). The variables IOP (*P* < 0.001) and age (*P* < 0.001) were not normally distributed and as such, the relationship between IOP and age was examined using Spearman’s correlation coefficient. No correlation was observed between IOP and age (rho = −0.14, *P* = 0.095). Average IOP was not significantly different between male and female dogs (*P* = 0.34).

### Dandie Dinmont terrier (DDT)

Of the 95 DDT examined, 35 (36.8 %) were male and 60 (63.2 %) were female*.* The median age of DDT examined was 47.52 months (minimum = 4.9 months, maximum = 165.27 months). Fifty two of 95 (54.7 %) DDT were affected by PLD; 31 (32.6 %) mildly affected (grade 1), 20 (21.1 %) moderately affected (grade 2) and 1 (1.1 %) severely affected (grade 3) (Table 1). A positive linear relationship was observed between PLD and age (rho = 0.57, *P* < 0.001). A significant negative correlation was observed between PLD and IOP (rho = −0.35, *P* = 0.001). The proportion of dogs affected by PLD (grades 1–3) was not significantly different between male and female dogs (*P* = 0.86). The variables IOP (*P* = 0.01) and age (*P* < 0.001) were not normally distributed and as such, the relationship between IOP and age was examined using Spearman’s correlation coefficient. A moderately strong and significant negative correlation was observed between IOP and age in DDT dogs (rho = − 0.578, *P* < 0.0001).

### Interbreed comparison

The prevalence of PLD was significantly higher in BH (145/198) compared to FCR (106/170, *P* = 0.025) or DDT (52/95, *P* = 0.002). No significant difference was observed in the distribution of age and sex among the three dog breeds (Table 2). However, average IOP among the three dog breeds was significantly different (*P* < 0.001; Table 2). Average IOP in DDT was significantly higher compared to BH (*P* = 0.039) or FCR (*P* < 0.001).

**Table 2:** Comparison of distribution of mean age, sex, and mean IOP in BH, FCR and DDT dogs

Variable	Breed			
	BH	FCR	DDT	*P* value
Age at examination				
Median (Months)	43.07	48.25	47.52	0.20
Sex				
Male	72 (36.4 %)	67 (39 %)	35 (36.8 %)	0.82
Female	126 (63.6 %)	103 (61 %)	60 (63.2 %)	
Intraocular pressure				
Median (mmHg)	13.50	12.50	14.17	<0.001

## Discussion

This study provides current prevalence data for PLD in the United Kingdom populations of BH, FCR and DDT. We made considerable efforts to recruit BH, FCR and DDT onto our study that were representative of the UK populations of these breeds. Gonioscopy screening sessions were undertaken in different locations around the UK and at different types of event, including dog shows, ‘fun days’ and breed information days. The gonioscopy screening was promoted by a variety of different mechanisms, including correspondence from the Kennel Club to the owners of Kennel Club registered dogs of each breed, via breed club websites and via social media. All dogs that were volunteered for screening were accepted, regardless of their age, ancestry or Kennel Club registration status. While we cannot demonstrate conclusively that the dogs in our study are truly representative of the UK populations of BH, FCR and DDT we have used recruitment methods equivalent to those used by others to report the prevalence of PLD in other UK populations of dogs [13, 16]. By including dogs of varying ages we have avoided the potential bias that may be associated with estimating the prevalence of ocular disorders using data from recognised eye screening schemes, such as the BVA/KC/ISDS or ECVO schemes, the majority of which is derived from young dogs [17].

Previous PLD prevalence data for the BH and DDT are lacking but prevalence of PLD in the FCR was reported by Read et al. in 1998 [16]. Read et al. reported 34.7 % FCR to be moderately or severely affected by PLD which is much higher than the 21.2 % we report here. The most likely explanation for the reduction in prevalence of PLD in this breed is increased awareness of the anomaly amongst FCR breeders since the initial report and subsequent increased uptake in gonioscopic screening of dogs before breeding with exclusion of affected individuals from the breeding population. Another possible explanation for the difference prevalence rates is differences in age between the two groups. PLD has been shown to be progressive in this breed and Wood et al. also found a significant positive association between PLD and age [3, 13]. This seems an unlikely explanation, however, as, although Wood et al. did not report mean age of the population they investigated, 77.6 % of the dogs studied were < 60 months when examined whereas the mean age of our population was 58.7 months.

Our study revealed 38.4 % BH to be affected by moderate or severe PLD which was significantly higher compared to FCR (21.2 %) and DDT (22.1 %). The explanation for the higher PLD prevalence in the BH is most likely explained by a higher frequency of the genetic factor(s) responsible for PLD in this breed which could be a result of the use of popular sires that carry these factors and/or a reduced uptake of gonioscopic screening within this breed.

A significant positive linear relationship was observed between PLD and age in each of the three breeds studied here. Age-related narrowing of the ICA has been a generally accepted phenomenon for some time but only recently has PLD been formally recognised as progressive when Pearl et al. reported that 39 of 96 (40.6 %) FCR demonstrated PLD progression over time [5, 13, 14]. Because an association with age would not be expected for a congenital, non-progressive disorder our results thus both support the previous finding in the FCR and provide cross-sectional evidence of a similar progressive nature of PLD in other breeds.

The prevalence of PLD, a consistent risk factor for canine PCAG, between the two sexes appears to vary with breed. Our study showed that female BH were more likely to have PLD than male BH, but the same was not true of the FCR and DDT breeds. A sex predisposition for goniodysgenesis/PLD has previously been reported in the American Cocker spaniel but not in the English springer spaniel, FCR or Samoyed [6, 8, 15, 18]. The differences between the breeds might be explained by, as yet, undetermined genetic factors and the breed differences we have observed suggest genetic risk factors for PLD might well vary between the breeds included in our study. In man, females are affected by PCAG more frequently than males [8–10]. This is likely due to differences in anterior chamber morphology between the sexes. A shallow anterior chamber is the major risk factor for development of angle closure glaucoma in man, and women have shallower anterior chambers than men [19–21]. The effect of sex on PCAG in dogs seems to vary with breed. A sex predisposition has been reported in American Cocker spaniels, Cocker spaniels, BH, Welsh springer spaniels and Samoyeds [2, 11]. Currently, there is no clear explanation for a predisposition to PCAG in female dogs which is consistent between breeds. In Beagles, a breed not typically considered to be affected by PCAG, females have been shown to have differences in anterior chamber morphology compared to males [18]. The angle opening distance (the perpendicular distance from the end of Descemet’s membrane to the anterior iris) was significantly smaller for female dogs than male dogs [18]. However, in the Samoyed and English springer spaniel, breeds known to be affected by PCAG, there was no difference between width of the ciliary cleft between female and male dogs [5, 14].

We also investigated possible associations between IOP and PLD, age and sex in the three breeds included in our study. IOP is known to be influenced by a number of factors in dogs including diurnal variations and age. To reduce the possible influence of diurnal variations in our study, all tonometric measurements were performed between the hours of 10:00 and 16:00 GMT. An association of IOP with age has already been reported in the Samoyed [14]. In this breed, a significantly higher IOP was demonstrated in dogs less than 1 year old compared to older dogs and, in animals 7 years or older, there was a pronounced decrease in IOP. Our study failed to show any association between IOP and age in the BH or FCR. However, in the DDT, a moderately strong negative correlation was observed between IOP and age. The reason for this finding is unknown.

Previous studies have shown no association between IOP and the degree of PLD in the FCR and English springer spaniel [3, 5]. We demonstrated a similar lack of association in the FCR and BH. However, we did find a significant association between IOP and degree of PLD in the DDT. The median IOP decreased gradually with increasing degree of PLD (i.e., grades 0, 1, 2, 3). The reasons for the different findings in the DDT are, as yet, unknown. Explanations may lie in differences in the anatomy of the ICA between the different breeds which, in turn, may relate to genetic variations. It is our aim to further characterize ICA anatomy with the use of ultrasound biomicroscopy in dogs affected and unaffected by PLD of these, and other, breeds. We have also collected DNA from all the dogs included in our study and are in the process of performing genome wide association studies in these breeds to look for possible associations between PLD and genetic variations both within and between breeds.

## Conclusions

We have provided prevalence data for PLD in the BH, FCR and DDT. Overall PLD prevalence remains high in all three breeds which is most likely explained by progression of PLD over time. The associations between PLD and sex in the BH and between IOP and both PLD and age in the DDT are novel findings and warrant further investigation.

## Methods

### Study design and population

This study was designed as a cross sectional study. Three different breeds of dogs were enrolled during gonioscopy screening sessions at different locations across the United Kingdom between September 2013 and December 2014 between the hours of 10:00 and 16:00 GMT. To the best of our ability, we recruited BH, FCR and DDT onto our study that were representative of the UK populations of these breeds. Gonioscopy screening sessions were undertaken in different locations around the UK and at different types of event, including dog shows, ‘fun days’ and breed information days. The gonioscopy screening was promoted by a variety of different mechanisms, including correspondence from the Kennel Club to the owners of Kennel Club registered dogs of each breed, via breed club websites and via social media. All dogs that were volunteered for screening were accepted, regardless of their age, ancestry or Kennel Club registration status. Dogs enrolled in the study included: 198 Basset hounds (BH), 170 Flatcoated retrievers (FCR) and 95 Dandie Dinmont terriers (DDT). For each dog, data were collected on sex and age at time of examination. All experiments were conducted in accordance with the ARVO statement for the Use of Animals in Ophthalmic and Vision Research and approved by the Animal Health Trust’s Research and Ethical Approval Committee. All dogs were pets and ophthalmological examination was only performed after informed and written owner consent.

### Examination procedure

**Fig. 1:**
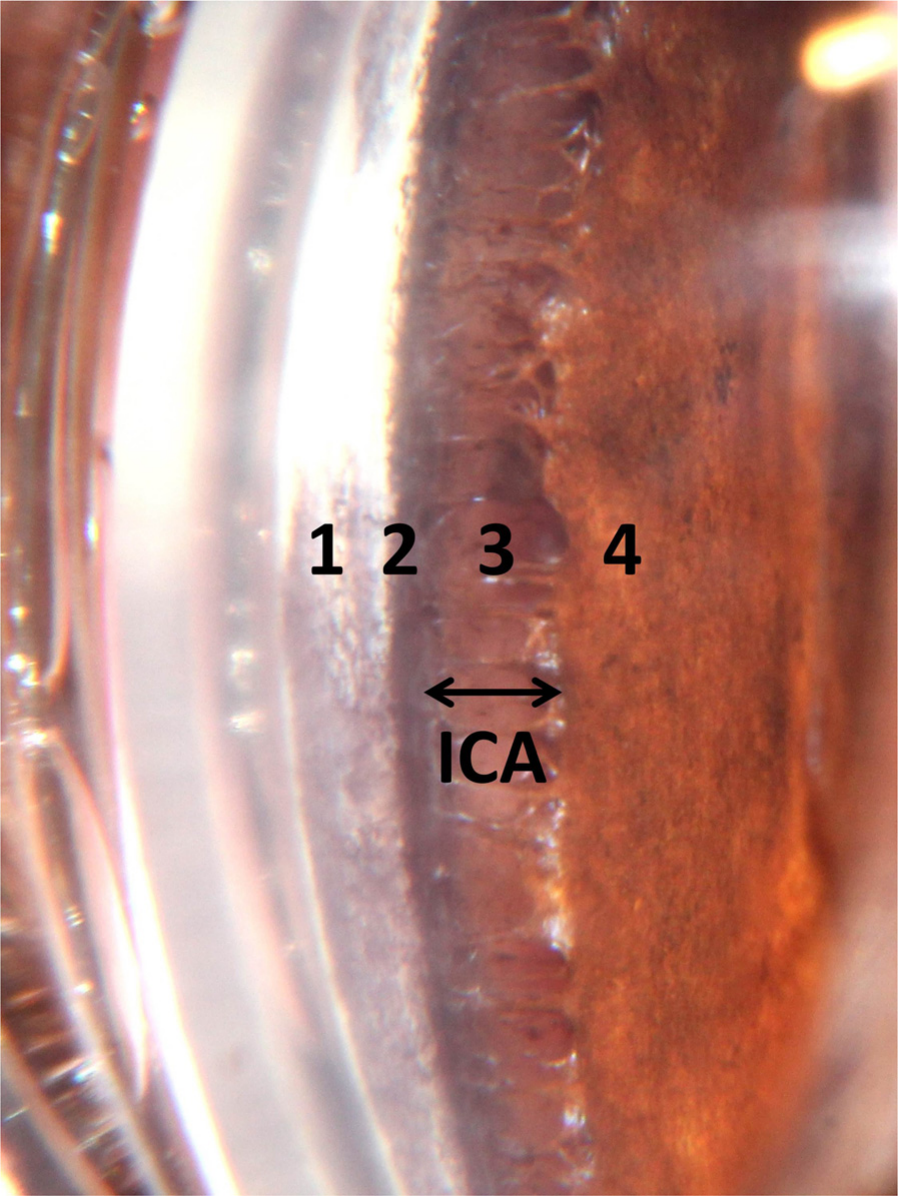
The gonioscopic view of a normal canine iridocorneal angle (ICA). 1 = corneoscleral limbus, 2 = site of insertion of pectinate ligament fibres, 3 = pectinate ligament fibres overlying ciliary cleft and 4 = iris. The double-headed black arrow delineates the opening of the ICA

**Fig. 2:**
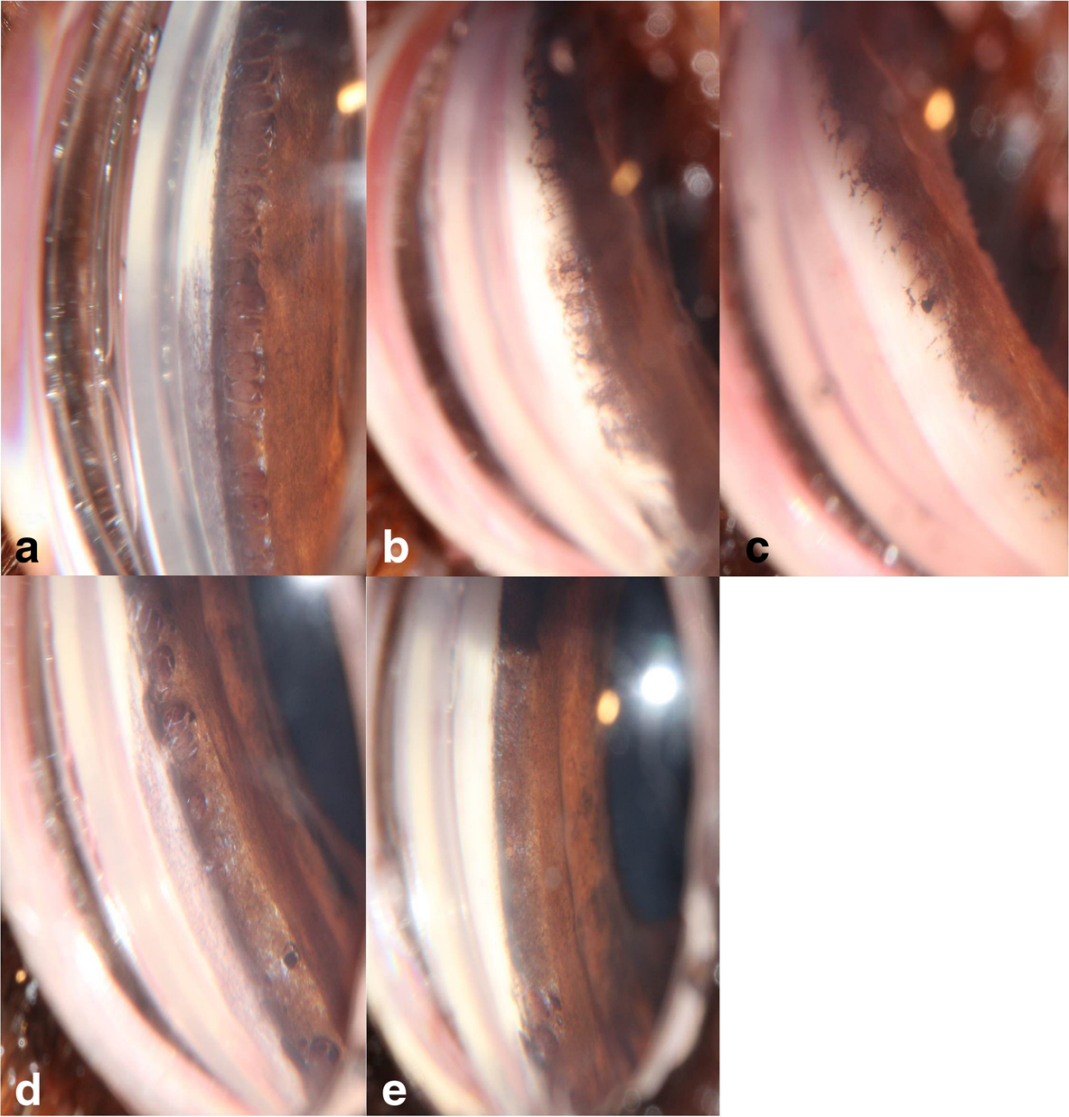
A series of gonioscopy photographs to illustrate the pectinate ligament dysplasia (PLD) grading system used. The grading system has been applied assuming the photographs are representative of the entire 360° of iridocorneal angle (ICA) in each case. **a** Normal appearance of the ICA and pectinate ligament. Unaffected (grade 0). **b** Approximately 15 % of the ICA is affected by abnormally thickened pectinate ligament fibres. Mildly affected (grade 1). **c** Approximately 25 % of the ICA is affected by pectinate ligament ‘sheeting’. Moderately affected (grade 2). **d** Approximately 75 % of the ICA is affected by pectinate ligament ‘sheeting’. Moderately affected (grade 2). **e** Over 90 % of the ICA is affected by ‘sheeting’. Severely affected (grade 3

Gonioscopy was performed bilaterally in conscious dogs following application of 0.5 % proxymetacaine (Bausch & Lomb, Chauvin Pharmaceuticals Ltd., Aubenas, France) using a 17 or 19 mm Koeppe goniolens (Ocular Instruments, Redmond, Washington, USA) filled with 2 mg/g carbomer gel (Viscotears; Alcon, Hemel Hempstead, UK) before placing onto the cornea. The entire 360° of the ICA was then examined using a handheld slit-lamp biomicroscope (Keeler PSL Classic, Berkshire, UK) for the presence of PLD which was quantified according to the percentage of the ICA circumference affected, estimating this to the nearest 5 %. Regions of ICA were judged to be affected by PLD where they exhibited abnormally broad pectinate ligament fibers or solid sheets of tissue as previously described (Figs. 1 and 2) [13, 16]. No attempt to measure ICA width was made. The same examiner, a diplomate of the European College of Veterinary Ophthalmologists (JO) performed all the examinations to reduce the influence of subjectivity. Of the dogs examined gonioscopically, IOP was estimated using rebound tonometry (TonoVet, Kruuse, Langescov, Denmark) in 198 BH, 137 FCR and 95 DDT. Tonometry was performed immediately prior to topical anesthesia and gonioscopy. This tonometer takes six consecutive measurements, discards the highest and lowest readings and averages the remaining four. Only readings with a maximum standard deviation of 5 % were noted. Three averaged readings were taken for each eye and the average of these three readings was then calculated.

### Statistical analyses

To reduce the influence of subjectivity of measurement of PLD (percentage of ICA circumference affected), PLD results were assigned to one of 4 ordinal scale grades, based on the percentage of ICA circumference affected (Table 3) as previously described [13]. Eyes were classified as ‘unaffected’ if 0 % of the ICA was affected by PLD (ordinal grade 0), ‘mildly affected’ if < 20 % was affected (grade 1), ‘moderately affected’ if 20–90 % was affected (grade 2) and ‘severely affected’ if > 90 % was affected (grade 3) (Fig. 2). The mean percentage of ICA affected by PLD and mean IOP for the left and right eyes were averaged for each dog, and the averaged values used in subsequent analyses. For each breed, the prevalence of PLD was determined by calculating proportions of dogs graded as 0, 1, 2, and 3. The linear relationships between the ordinal variable PLD (grade 0, 1, 2, 3) and age and PLD (grade 0, 1, 2, 3) and IOP were evaluated using Spearman’s correlation coefficient (rho) and prior to this analysis, the continuous variables age and IOP were converted into ordinal variables with 4 ranks based on quartile values. The proportions of male and female dogs affected with PLD (grade 0, 1, 2, 3) were compared using the Chi-square test of independence. The linear relationship between IOP and age was assessed using Spearman’s correlation coefficient and prior to this analysis, the variables IOP and age were examined for normal distribution using Shapiro-Wilk test, and the overall pattern of the data and deviations from the pattern were examined using scatterplots. The variables IOP and age were not normally distributed for the three breeds, and therefore the linear relationship between IOP and age was assessed using Spearman’s correlation coefficient. Prior to this analysis the continuous variables age and IOP were each converted into ordinal variables with 4 ranks based on quartile values. The difference in average IOP between male and female dogs was assessed using the Mann–Whitney *U* test. Comparisons among the three breeds (BH, FCR, and DDT) were performed for the variables age and IOP using the Kruskal-Wallis test. The proportions of male and female dogs in the three breeds were compared using the Chi-square test of independence. A *P* value of < 0.05 was considered statistically significant. All analyses were performed using IBM SPSS statistical software (version 22).

**Table 3:** Degree of PLD estimated by gonioscopy as percentage of total ICA circumference affected and equivalent ordinal grade as published by Pearl et al. [21]

Percentage of ICA affected	Definition	Ordinal grade
0 %	Unaffected	0
<20 %	Mildly affected	1
20–90 %	Moderately affected	2
>90 %	Severely affected	3
